# 2-[(*E*)-(2,4,6-Trichloro­phen­yl)imino­meth­yl]phenol

**DOI:** 10.1107/S1600536811026122

**Published:** 2011-07-06

**Authors:** Hoong-Kun Fun, Ching Kheng Quah, S. Viveka, D. J. Madhukumar, G. K. Nagaraja

**Affiliations:** a X-ray Crystallography Unit, School of Physics, Universiti Sains Malaysia, 11800 USM, Penang, Malaysia; b Department of Chemistry, Mangalore University, Karnataka, India

## Abstract

The title mol­ecule, C_13_H_8_Cl_3_NO, exists in a *trans* configuration with respect to the C=N bond [1.278 (2) Å]. The benzene rings form a dihedral angle of 24.64 (11)°. The mol­ecular structure is stabilized by an intra­molecular O—H⋯N hydrogen bond, which generates an *S*(6) ring motif. In the crystal, π–π stacking inter­actions [centroid–centroid distances = 3.6893 (14) Å] are observed.

## Related literature

For general background to and the pharmacological activity of Schiff base compounds, see: Shapiro (1998 ▶); Villar *et al.* (2004 ▶); Venugopal & Jayashree (2008 ▶); Pandey *et al.* (2003 ▶); Bhat *et al.* (2005 ▶); Wadher *et al.* (2009 ▶). For related structures, see: Fun *et al.* (2011*a* ▶,*b* ▶). For hydrogen-bond motifs, see: Bernstein *et al.* (1995 ▶). For standard bond-length data, see: Allen *et al.* (1987 ▶).
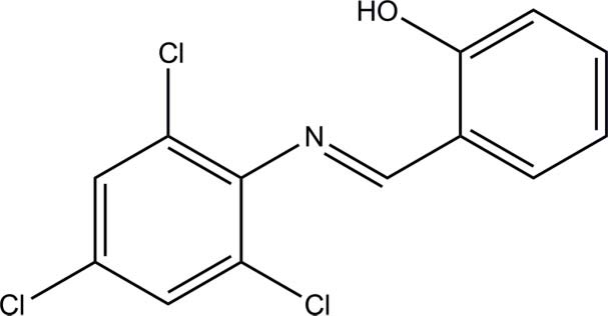


## Experimental

### 

#### Crystal data

C_13_H_8_Cl_3_NO
*M* *_r_* = 300.55
Monoclinic, 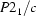

*a* = 12.8847 (16) Å
*b* = 6.9505 (9) Å
*c* = 14.4265 (18) Å
β = 96.612 (2)°
*V* = 1283.4 (3) Å^3^
*Z* = 4
Mo *K*α radiation
μ = 0.70 mm^−1^
*T* = 296 K
0.36 × 0.19 × 0.14 mm

#### Data collection

Bruker SMART APEXII DUO CCD area-detector diffractometer
Absorption correction: multi-scan (*SADABS*; Bruker, 2009 ▶) *T*_min_ = 0.785, *T*_max_ = 0.908
10136 measured reflections
3782 independent reflections
2586 reflections with *I* > 2σ(*I*)
*R*_int_ = 0.027

#### Refinement

*R*[*F* ^2^ > 2σ(*F* ^2^)] = 0.043
*wR*(*F* ^2^) = 0.124
*S* = 1.02
3782 reflections
167 parameters
H atoms treated by a mixture of independent and constrained refinement
Δρ_max_ = 0.45 e Å^−3^
Δρ_min_ = −0.44 e Å^−3^

### 

Data collection: *APEX2* (Bruker, 2009 ▶); cell refinement: *SAINT* (Bruker, 2009 ▶); data reduction: *SAINT*; program(s) used to solve structure: *SHELXTL* (Sheldrick, 2008 ▶); program(s) used to refine structure: *SHELXTL*; molecular graphics: *SHELXTL*; software used to prepare material for publication: *SHELXTL* and *PLATON* (Spek, 2009 ▶).

## Supplementary Material

Crystal structure: contains datablock(s) global, I. DOI: 10.1107/S1600536811026122/lh5276sup1.cif

Structure factors: contains datablock(s) I. DOI: 10.1107/S1600536811026122/lh5276Isup2.hkl

Supplementary material file. DOI: 10.1107/S1600536811026122/lh5276Isup3.cml

Additional supplementary materials:  crystallographic information; 3D view; checkCIF report

## Figures and Tables

**Table 1:** Hydrogen-bond geometry (Å, °)

*D*—H⋯*A*	*D*—H	H⋯*A*	*D*⋯*A*	*D*—H⋯*A*
O1—H1*O*1⋯N1	0.79 (3)	1.94 (3)	2.633 (2)	146 (3)

**Fig. 1.**
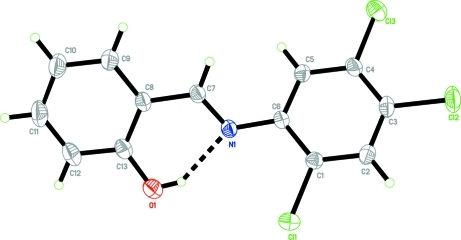
The molecular structure of the title compound showing 30% probability displacement ellipsoids for non-H atoms. An intramolecular hydrogen bond is shown as a dashed line.

## supplementary crystallographic information

### Comment

Schiff bases are the important compound owing to their wide range of biological activities and industrial application. The synthesis and structural research of Schiff bases are derived from aldehydes and amines bearing various alkyl and aryl *N*-substituents. Schiff base ligands may contain a variety of substituents with different electron-donating or electron-withdrawing groups and therefore may have interesting chemical properties. They have attracted particular interest due to their biological activities (Shapiro, 1998). They have been found to posses the pharmacological activities such as antimalarial, anticancer (Villar *et al.*, 2004) antibacterial (Venugopal & Jayashree, 2008) antifungal (Pandey *et al.*, 2003) antitubercular (Bhat *et al.*, 2005), anti-inflammatory and antimicrobial (Wadher *et al.*, 2009) properties.

In the title molecule (Fig. 1), the benzene rings (C1-C6 and C8-C13) form a dihedral angle of 24.64 (11)°. The title molecule exists in *trans* configuration with respect to the  C7═N1 bond [C7═N1 = 1.278 (2) Å]. Bond lengths (Allen *et al.*, 1987) and angles are within normal ranges and are comparable to related structures (Fun *et al.*, 2011*a*,*b*). The molecular structure is stabilized by an intramolecular O1–H1O1···N1 hydrogen bond (Table 1) which generates a *S*(6) ring motif (Fig. 1, Bernstein *et al.*, 1995).

In the crystal packing, π-π stacking interactions between the centroid of C1-C6 (Cg1) and C8-C13 (Cg2) benzene rings, with Cg1···Cg2^i^ distance of 3.6893 (14) Å [symmetry code: (i) -x, -1/2+y, 3/2-z] are observed. No significant intermolecular hydrogen bonds are observed.

### Experimental

A mixture of salicylaldehyde (0.01 mol) and 2,4,6-trichloroaniline (0.01 mol) was dissolved in a minimum amount of ethanol, followed by addition of 2 mL glacial acetic acid. The mixture was refluxed gently for 4-5 h. The reaction was monitored by TLC. After completion of the reaction, the mixture was poured into a beaker containing crushed ice. The precipitate obtained was filtered, dried and recrystallized from ethanol. Yield: 68%, *m.p.* 425-426 K.

### Refinement

H1O1 atom was located in a difference Fourier map and refined freely [O1–H1O1 = 0.79 (3) Å]. The remaining H atoms were positioned geometrically and refined using a riding model with C–H = 0.93 Å and *U*_iso_(H) = 1.2 *U*_eq_(C). The highest residual electron density peak and the deepest hole are located at 0.93 and 0.85 Å from Cl3, respectively.

### Crystal data

**Table:**

C_13_H_8_Cl_3_NO	*F*(000) = 608
*M**_r_* = 300.55	*D*_x_ = 1.556 Mg m^−^^3^
Monoclinic, *P*2_1_/*c*	Mo *K*α radiation, λ = 0.71073 Å
Hall symbol: -P 2ybc	Cell parameters from 3223 reflections
*a* = 12.8847 (16) Å	θ = 2.9–30.0°
*b* = 6.9505 (9) Å	µ = 0.70 mm^−^^1^
*c* = 14.4265 (18) Å	*T* = 296 K
β = 96.612 (2)°	Block, yellow
*V* = 1283.4 (3) Å^3^	0.36 × 0.19 × 0.14 mm
*Z* = 4	

### Data collection

**Table:**

Bruker SMART APEXII DUO CCD area-detector diffractometer	3782 independent reflections
Radiation source: fine-focus sealed tube	2586 reflections with *I* > 2σ(*I*)
graphite	*R*_int_ = 0.027
φ and ω scans	θ_max_ = 30.2°, θ_min_ = 3.1°
Absorption correction: multi-scan (*SADABS*; Bruker, 2009)	*h* = −18→18
*T*_min_ = 0.785, *T*_max_ = 0.908	*k* = −9→9
10136 measured reflections	*l* = −20→20

### Refinement

**Table:**

Refinement on *F*^2^	Primary atom site location: structure-invariant direct methods
Least-squares matrix: full	Secondary atom site location: difference Fourier map
*R*[*F*^2^ > 2σ(*F*^2^)] = 0.043	Hydrogen site location: inferred from neighbouring sites
*wR*(*F*^2^) = 0.124	H atoms treated by a mixture of independent and constrained refinement
*S* = 1.02	*w* = 1/[σ^2^(*F*_o_^2^) + (0.0489*P*)^2^ + 0.6136*P*] where *P* = (*F*_o_^2^ + 2*F*_c_^2^)/3
3782 reflections	(Δ/σ)_max_ < 0.001
167 parameters	Δρ_max_ = 0.45 e Å^−^^3^
0 restraints	Δρ_min_ = −0.44 e Å^−^^3^

### Special details

**Table:**

Geometry. All esds (except the esd in the dihedral angle between two l.s. planes) are estimated using the full covariance matrix. The cell esds are taken into account individually in the estimation of esds in distances, angles and torsion angles; correlations between esds in cell parameters are only used when they are defined by crystal symmetry. An approximate (isotropic) treatment of cell esds is used for estimating esds involving l.s. planes.
Refinement. Refinement of F^2^ against ALL reflections. The weighted R-factor wR and goodness of fit S are based on F^2^, conventional R-factors R are based on F, with F set to zero for negative F^2^. The threshold expression of F^2^ > 2sigma(F^2^) is used only for calculating R-factors(gt) etc. and is not relevant to the choice of reflections for refinement. R-factors based on F^2^ are statistically about twice as large as those based on F, and R- factors based on ALL data will be even larger.

### Fractional atomic coordinates and isotropic or equivalent isotropic displacement parameters (Å^2^)

**Table:**

	*x*	*y*	*z*	*U*_iso_*/*U*_eq_	
Cl1	0.05175 (4)	0.34050 (9)	0.92951 (3)	0.05264 (16)	
Cl2	0.44455 (4)	0.27949 (11)	0.84597 (5)	0.0669 (2)	
Cl3	0.35845 (4)	0.16620 (13)	0.63858 (4)	0.0723 (2)	
O1	−0.20343 (13)	0.2343 (3)	0.77771 (11)	0.0562 (4)	
N1	−0.01212 (11)	0.2621 (2)	0.73161 (11)	0.0370 (3)	
C1	0.13702 (14)	0.3001 (3)	0.84736 (13)	0.0368 (4)	
C2	0.24358 (14)	0.3061 (3)	0.87539 (14)	0.0427 (4)	
H2A	0.2685	0.3344	0.9369	0.051*	
C3	0.31237 (14)	0.2693 (3)	0.81056 (15)	0.0428 (4)	
C4	0.27450 (14)	0.2244 (3)	0.71929 (14)	0.0427 (4)	
C5	0.16785 (14)	0.2223 (3)	0.69162 (13)	0.0408 (4)	
H5A	0.1432	0.1931	0.6301	0.049*	
C6	0.09726 (13)	0.2636 (3)	0.75513 (12)	0.0346 (4)	
C7	−0.05235 (13)	0.3079 (3)	0.64944 (13)	0.0372 (4)	
H7A	−0.0084	0.3437	0.6056	0.045*	
C8	−0.16440 (14)	0.3060 (3)	0.62241 (14)	0.0387 (4)	
C9	−0.20251 (16)	0.3408 (4)	0.52901 (15)	0.0514 (5)	
H9A	−0.1558	0.3640	0.4857	0.062*	
C10	−0.30845 (17)	0.3411 (4)	0.50043 (18)	0.0628 (7)	
H10A	−0.3333	0.3623	0.4382	0.075*	
C11	−0.37712 (17)	0.3093 (4)	0.56579 (19)	0.0638 (7)	
H11A	−0.4486	0.3109	0.5471	0.077*	
C12	−0.34176 (16)	0.2754 (4)	0.65778 (19)	0.0561 (6)	
H12A	−0.3893	0.2549	0.7006	0.067*	
C13	−0.23490 (15)	0.2716 (3)	0.68736 (15)	0.0430 (4)	
H1O1	−0.142 (2)	0.238 (5)	0.787 (2)	0.077 (10)*	

### Atomic displacement parameters (Å^2^)

**Table:**

	*U*^11^	*U*^22^	*U*^33^	*U*^12^	*U*^13^	*U*^23^
Cl1	0.0521 (3)	0.0677 (4)	0.0399 (2)	0.0004 (3)	0.0130 (2)	−0.0002 (2)
Cl2	0.0322 (2)	0.0865 (5)	0.0786 (4)	−0.0046 (3)	−0.0083 (2)	0.0036 (3)
Cl3	0.0415 (3)	0.1152 (6)	0.0624 (4)	0.0192 (3)	0.0154 (2)	−0.0023 (4)
O1	0.0452 (8)	0.0715 (12)	0.0537 (9)	0.0035 (8)	0.0128 (7)	0.0064 (8)
N1	0.0303 (7)	0.0408 (9)	0.0400 (8)	0.0040 (6)	0.0037 (6)	−0.0019 (7)
C1	0.0366 (8)	0.0376 (10)	0.0365 (8)	−0.0005 (8)	0.0054 (7)	0.0016 (8)
C2	0.0388 (9)	0.0464 (12)	0.0407 (9)	−0.0037 (8)	−0.0044 (7)	0.0007 (8)
C3	0.0300 (8)	0.0448 (11)	0.0518 (11)	−0.0015 (8)	−0.0036 (7)	0.0060 (9)
C4	0.0319 (8)	0.0509 (12)	0.0459 (10)	0.0077 (8)	0.0070 (7)	0.0037 (9)
C5	0.0337 (8)	0.0502 (12)	0.0379 (9)	0.0065 (8)	0.0012 (7)	−0.0009 (8)
C6	0.0291 (7)	0.0349 (9)	0.0396 (9)	0.0009 (7)	0.0032 (6)	0.0000 (7)
C7	0.0309 (8)	0.0399 (10)	0.0411 (9)	0.0031 (7)	0.0049 (7)	−0.0029 (8)
C8	0.0305 (8)	0.0391 (10)	0.0460 (9)	0.0031 (7)	0.0020 (7)	−0.0049 (8)
C9	0.0404 (10)	0.0649 (15)	0.0473 (10)	0.0039 (10)	−0.0016 (8)	−0.0046 (10)
C10	0.0443 (11)	0.0808 (19)	0.0592 (13)	0.0061 (11)	−0.0114 (10)	−0.0079 (13)
C11	0.0352 (10)	0.0735 (18)	0.0790 (17)	0.0022 (11)	−0.0098 (10)	−0.0134 (14)
C12	0.0346 (9)	0.0597 (15)	0.0752 (15)	−0.0016 (10)	0.0109 (10)	−0.0075 (12)
C13	0.0360 (9)	0.0391 (11)	0.0544 (11)	0.0025 (8)	0.0072 (8)	−0.0047 (9)

### Geometric parameters (Å, °)

**Table:**

Cl1—C1	1.7292 (19)	C5—H5A	0.9300
Cl2—C3	1.7222 (18)	C7—C8	1.452 (2)
Cl3—C4	1.726 (2)	C7—H7A	0.9300
O1—C13	1.345 (3)	C8—C13	1.399 (3)
O1—H1O1	0.79 (3)	C8—C9	1.401 (3)
N1—C7	1.278 (2)	C9—C10	1.380 (3)
N1—C6	1.411 (2)	C9—H9A	0.9300
C1—C2	1.387 (2)	C10—C11	1.383 (4)
C1—C6	1.393 (2)	C10—H10A	0.9300
C2—C3	1.384 (3)	C11—C12	1.373 (4)
C2—H2A	0.9300	C11—H11A	0.9300
C3—C4	1.386 (3)	C12—C13	1.394 (3)
C4—C5	1.386 (2)	C12—H12A	0.9300
C5—C6	1.393 (2)		
			
C13—O1—H1O1	110 (2)	N1—C7—H7A	119.0
C7—N1—C6	120.55 (16)	C8—C7—H7A	119.0
C2—C1—C6	121.80 (17)	C13—C8—C9	119.42 (18)
C2—C1—Cl1	118.76 (14)	C13—C8—C7	121.61 (18)
C6—C1—Cl1	119.44 (13)	C9—C8—C7	118.97 (18)
C3—C2—C1	119.14 (17)	C10—C9—C8	120.8 (2)
C3—C2—H2A	120.4	C10—C9—H9A	119.6
C1—C2—H2A	120.4	C8—C9—H9A	119.6
C2—C3—C4	120.04 (17)	C9—C10—C11	119.0 (2)
C2—C3—Cl2	118.70 (15)	C9—C10—H10A	120.5
C4—C3—Cl2	121.26 (16)	C11—C10—H10A	120.5
C3—C4—C5	120.32 (18)	C12—C11—C10	121.3 (2)
C3—C4—Cl3	120.94 (15)	C12—C11—H11A	119.4
C5—C4—Cl3	118.73 (15)	C10—C11—H11A	119.4
C4—C5—C6	120.60 (17)	C11—C12—C13	120.4 (2)
C4—C5—H5A	119.7	C11—C12—H12A	119.8
C6—C5—H5A	119.7	C13—C12—H12A	119.8
C1—C6—C5	118.01 (16)	O1—C13—C12	118.5 (2)
C1—C6—N1	118.57 (16)	O1—C13—C8	122.39 (18)
C5—C6—N1	123.37 (16)	C12—C13—C8	119.1 (2)
N1—C7—C8	122.10 (17)		
			
C6—C1—C2—C3	−1.9 (3)	C7—N1—C6—C1	151.17 (19)
Cl1—C1—C2—C3	178.45 (16)	C7—N1—C6—C5	−31.6 (3)
C1—C2—C3—C4	−0.9 (3)	C6—N1—C7—C8	179.28 (17)
C1—C2—C3—Cl2	179.37 (16)	N1—C7—C8—C13	6.2 (3)
C2—C3—C4—C5	2.1 (3)	N1—C7—C8—C9	−174.2 (2)
Cl2—C3—C4—C5	−178.18 (17)	C13—C8—C9—C10	−0.2 (4)
C2—C3—C4—Cl3	−176.72 (17)	C7—C8—C9—C10	−179.7 (2)
Cl2—C3—C4—Cl3	3.0 (3)	C8—C9—C10—C11	1.0 (4)
C3—C4—C5—C6	−0.5 (3)	C9—C10—C11—C12	−0.8 (4)
Cl3—C4—C5—C6	178.34 (16)	C10—C11—C12—C13	−0.3 (4)
C2—C1—C6—C5	3.4 (3)	C11—C12—C13—O1	−178.8 (2)
Cl1—C1—C6—C5	−176.92 (15)	C11—C12—C13—C8	1.1 (4)
C2—C1—C6—N1	−179.22 (18)	C9—C8—C13—O1	179.0 (2)
Cl1—C1—C6—N1	0.5 (3)	C7—C8—C13—O1	−1.5 (3)
C4—C5—C6—C1	−2.2 (3)	C9—C8—C13—C12	−0.9 (3)
C4—C5—C6—N1	−179.41 (19)	C7—C8—C13—C12	178.7 (2)

### Hydrogen-bond geometry (Å, °)

**Table:**

*D*—H···*A*	*D*—H	H···*A*	*D*···*A*	*D*—H···*A*
O1—H1O1···N1	0.79 (3)	1.94 (3)	2.633 (2)	146 (3)
